# Prevalence of risk factors for human immunodeficiency virus among women of reproductive age in Sierra Leone: a 2019 nationwide survey

**DOI:** 10.1186/s12879-022-07037-7

**Published:** 2022-01-17

**Authors:** Joseph Kawuki, Kassim Kamara, Quraish Sserwanja

**Affiliations:** 1 Centre for Health Behaviours Research, Jockey Club School of Public Health and Primary Care, The Chinese University of Hong Kong, Hong Kong, Hong Kong SAR, China; 2 Directorate of Health Security and Emergencies, Ministry of Health and Sanitation, Sierra Leone, Freetown, Sierra Leone; 3 Programmes Department, GOAL, Arkaweet Block 65 House No. 227, Khartoum, Sudan

**Keywords:** HIV/AIDS, Women, Risk-factors, Burden, DHS, Sierra Leone

## Abstract

**Background and aim:**

For over 40 years of the HIV/AIDS global epidemic, no effective cure nor vaccine is yet available, making the current control strategies focused on curbing new infections through risk reduction. The study aimed to determine the prevalence of HIV risk factors and their associated socio-demographics among women of reproductive age in Sierra Leone.

**Methods:**

We used weighted data from the Sierra Leone Demographic and Health Survey (SLDHS) of 2019 for 12,005 women aged 15–49 years. Multistage sampling was used to select study participants. Exposure to HIV risk factors was considered if a woman reported at least one of the following; having multiple sexual partners, transactional sex, non-condom use for the unmarried, and having other sexually transmitted infections (STIs). We, then, conducted multivariable logistic regression to explore the associated socio-demographics. All the analyses were done using SPSS (version 25).

**Results:**

Of the 12,005 women, 38.1% (4577/12005) (95% confidence interval (CI) 37.3–39.0) had at least one of the four risk factors. Women of 15 to 19 years (adjusted odds ratio (AOR) = 1.34, 95% CI 1.00–1.80) and 20 to 34 years (AOR = 1.25, 95% CI 1.05–1.49) had more odds of having HIV risk factors compared to those of 35 to 49 years. Urban residents (AOR = 1.49, 95% CI 1.17–1.89) and those from the Northwestern region (AOR = 1.81, 95% CI 1.26–2.60) were also more likely to encounter HIV risk factors compared to their respective counterparts. Moreover, unmarried women (AOR = 111.17, 95% CI 87.55–141.18) and those working (AOR = 1.38, 95% CI 1.14–1.67) also had higher odds of having HIV risk factors, compared to their respective counterparts. Sex of household head and parity were also significant associates.

**Conclusions:**

More than a third of women in Sierra Leone had encountered at least one HIV risk factor, and this was associated with age, place of residence, region, marital status, working status, household head and parity. There is a need for strengthening HIV/AIDS education programs, laws and policies targeting the young, working, unmarried and urban-resident women.

**Supplementary Information:**

The online version contains supplementary material available at 10.1186/s12879-022-07037-7.

## Introduction

Over 40 years since its identification, the human immunodeficiency virus (HIV) is still a global public health concern and one of the major causes of death, especially in developing countries [1]. According to a recent Joint United Nations Programme on HIV/AIDS (UNAIDS) report, over 37 million people globally were living with HIV in 2020, 1.5 million became infected and close to 1 million died due to acquired immunodeficiency syndrome (AIDS) related illnesses [1]. Africa still shares the biggest burden of HIV/AIDS, whereby over two-thirds of people living with HIV globally are found in sub-Saharan Africa [2].

In Sierra Leone, a small low-income West African country with a population size of around 8 million, the prevalence of HIV is around 1.7%, with a higher burden in Freetown- the capital [3–5]. Like in other countries, the key population groups in Sierra Leone include female sex workers, their partners and clients, long-distance truck drivers, members of the fishing community, men who have sex with men, transgender people, uniformed armed personnel, prisoners, and people who inject drugs (PWID), among others [3, 5]. Moreover, often the under-looked vulnerable group are the women; HIV/AIDS is shown to disproportionately affect women, where they account for more than half (55%) of people living with HIV/AIDS globally [2]. A similar trend has also been reported in Sierra Leone with women having a higher HIV prevalence than men (1.8% vs 1.3%) [6]. Besides the known risk of mother to child transmission, HIV/AIDS is also reported to have other adverse pregnancy complications [7] and is the leading cause of death among women of reproductive age [8]. In sub-Saharan Africa, the disproportionate burden of HIV/AIDS among women is attributed to lack of power, early marriages, coerced first sex, limited access to reproductive health services, extreme poverty, sexual violence, wars and conflict [9, 10].

Generally, several studies have explored the epidemiological characteristics of HIV/AIDS in Sierra Leone, with some studies focusing on HIV prevalence, testing and associated factors [6, 11–17], or other aspects [18]. These studies have focused mainly on the general population [6, 12, 15–18], military personnel [11], adolescents [13] and some other key population groups [14], but not women. Moreover, several factors/behaviours have been documented in Sierra Leone and elsewhere to increase the risk of HIV, which include; having multiple sexual partners, other sexually transmitted infections (STIs), inconsistent condom use, engaging in transactional sex, alcohol and drug use before/during sex, among others [11, 13, 19–22].

Given the considerable vulnerability of women to HIV/AIDS, we, therefore, aimed to determine the burden, in terms of prevalence, of HIV risk factors and their associated socio-demographics among women of reproductive age in Sierra Leone, using a nationwide dataset. We hypothesized that several socio-demographic characteristics are associated with HIV risk factors/behaviours. Thus establishing the magnitude of these risk factors and their associates would be vital in informing targeted interventions to address this global epidemic.

## Methods

### Study design and sampling methods

The Sierra Leone Demographic and Health Surveys (SLDHS) are cross-sectional surveys that are periodically conducted to obtain information on demographic, health and nutritional indicators of non-elderly adults and children. The latest survey was conducted over 4 months between May 2019 and August 2019 [5]. This national survey used a stratified, two-stage cluster sampling design with the first stage having 578 enumeration areas (EAs) (214 urban and 364 rural) selected leading to 13,872 households [5]. Using interviewer-administered questionnaires, the survey obtained sociodemographic information about the respondents. A detailed explanation of the sampling process is available elsewhere [5]. Women aged 15–49 years who were either permanent residents or visitors who had stayed in the selected households the night before the survey were eligible for interviews with a total of 15,574 women being interviewed. Of these, a weighted sample of 12,005 had been sexually active within 12 months preceding the survey and was included in this secondary analysis as shown in Table 1. Written informed consent was provided by all participants of the survey. Written permission to access the whole SLDHS database was obtained through the DHS program website [23].

**Table 1:** Socio-demographic characteristics of reproductive aged women in Sierra Leone as per the 2019 SLDHS

Characteristics	N = 12,005	%
Age		
15 to 19	1716	14.3
20 to 34	6216	51.8
35 to 49	4073	33.9
Visited by fieldworker		
No	8539	71.1
Yes	3466	28.9
Visited health facility		
No	5254	43.8
Yes	6751	56.2
Residence		
Rural	6654	55.4
Urban	5351	44.6
Region		
Western	2841	23.7
Southern	2260	18.8
Northwestern	1973	16.4
Northern	2587	21.5
Eastern	2345	19.5
Religion		
Islam	9193	76.6
Christianity and others	2812	23.4
Sex household head		
Male	8654	72.1
Female	3351	27.9
Household size		
7 and above	5230	43.6
Less than 7	6775	56.4
Working status		
Not working	3132	26.1
Working	8873	73.9
Marital status		
Married	8495	70.8
Not married	3510	29.2
Education level		
No education	5832	48.6
Primary education	1491	12.4
Secondary education	4112	34.3
Tertiary	570	4.8
Wealth index		
Poorest	2156	18.0
Poorer	2283	19.0
Middle	2307	19.2
Richer	2529	21.1
Richest	2730	22.7
Parity		
1–3	929	60.2
4 and above	615	39.8
Exposure to radio		
No	6471	53.9
Yes	5534	46.1
Exposure to newspapers		
No	11,052	92.1
Yes	953	7.9
Exposure to TV		
No	8652	72.1
Yes	3353	27.9
Internet use		
No	10,175	84.7
Yes	1830	15.3
Permission to access healthcare		
Big problem	2860	23.8
Not big problem	9146	76.2
Distance to health facility		
Big problem	5243	43.7
Not big problem	6762	56.3

### Variables of the study

#### Dependent variable

Four variables from the SLDHS were used in this study to measure the risk factors for HIV and these included; (1) engaging in sex with more than one partner in the past 12 months, (2) engaging in transactional sex in the past 12 months, (3) not using a condom during the most recent intercourse for those who were not married, and (4) having had a sexually transmitted infection in the past 12 months [19, 20]. The total number of risk factors per woman was not a variable in the SLDHS but was generated by first giving a score of 1 for every exposure to an HIV risk factor and a score of 0 for every non-exposure, and then summing up the scores for each woman. The minimum possible score is 0 while the maximum possible score is 4. Exposure to any of the four risk factors for HIV was categorized as a binary (Yes/No) outcome and women were considered to have been exposed to risk factors for HIV if they reported any of the four behaviours. The use of alcohol/being drunk during the last sexual intercourse was not included because the data was not available in the SLDHS data set.

#### Independent variables

Nineteen explanatory variables were used and included: Maternal age (15–19 years, 20–34 years and 35–49 years), Wealth index (poorest, poorer, middle, richer and richest quintiles), place of residence (urban and rural), region (Northern, Eastern, Southern, Western and Northwestern), level of education (no education, primary education, and post-primary education), household size (less than seven members and seven and above members), sex of household head (male or female), working status (not working and working), marital status (married including those in formal and informal unions, and not married). Religion was categorised as Muslims and Christians and others, problems seeking permission and distance to health facility were categorised as a big problem and no big problem while exposure to mass media was categorised as yes and no (if exposed to any of TV, radio, internet and newspapers), and visited by field worker or visited a health facility were categorised as yes and no, parity (less than 2, 2–4 and 5 and above). Wealth quintiles (poorest, poorer, middle, richer and richest) are a measure of relative household economic status and were calculated from household asset ownership information using Principal Component Analysis [5]. According to Sierra Leone, an urban area is a town with 2000 inhabitants or more [24].

### Statistical analysis

The study first presented descriptive statistics of the background characteristics and risk factors of HIV of the study sample. This study first examined the proportion of women that reported ever engaging in risk factors for HIV and then examined the factors associated with these risk factors. Bivariable and multivariable logistic regression analyses were conducted where variables found significant at p-value less than 0.25 in the bivariable analysis and not strongly collinear with other independent variables were included as candidate variables in the multivariable model [25]. Multi-collinearity was assessed using variance inflation factor (VIF) and wealth index was excluded in the multivariable model because it had VIFs above 3 with marital status, age, working, exposure to newspapers, internet, parity and problems with distance and seeking permission to healthcare. Hosmer and Lemeshow test was finally done to test the goodness of the multivariable regression model. Analysis was carried out based on the weighted count to account for the unequal probability sampling in different strata and to ensure the representativeness of the survey results at the national and regional levels. In order to account for the multi-stage cluster study design, we used SPSS (version 25.0) statistical software complex samples package incorporating the following variables in the analysis plan to account for the multistage sample design inherent in the DHS dataset: individual sample weight, sample strata for sampling errors/design, and cluster number [26–28]. Adjusted odds ratios (AOR), 95% confidence intervals (CI) and p-values were calculated with statistical significance level set at p-value < 0.05.

## Results

### Sociodemographic characteristics of study participants

In this study, a weighted sample of 12,005 women was used as shown in Table 1. The majority of women were aged 20–34 years (51.8%), had visited a health facility within 12 months preceding the survey (56.2%), working (73.9%), Muslims (76.6%), resided in rural areas (55.4%), no education (48.6%) and were married (70.8%). Most women had no exposure to mass media with 92.1%, 84.7%, 72.1% and 53.9% not being exposed to newspapers/magazines, internet, TV, and Radio respectively.

Out of the 12,005 women, 3289 (27.4%, 95% CI 26.6–28.2) were unmarried and had not used condom for their most recent sexual intercourse, 1490 (12.4%, 95% CI 11.9–13.1) had a sexually transmitted infection, 580 (4.8%, 95% CI 4.4–5.2) had multiple sexual partners, 154 (6.6%, 95% CI 5.5–7.4) had transactional sex and 4577 (38.1%, 95% CI 37.3–39.0) had any of the four risk factors as shown in Table 2. Additional file 1 shows how many women had one, two, three or four HIV risk factors.

**Table 2:** Prevalence of risk factors for HIV among reproductive age women in Sierra Leone

Risky factor	Frequency N = 12,005	Prevalence %	95% confidence interval
Non-condom use at last sexual intercourse for unmarried women	3289	27.4	26.6–28.2
Transactional sex^a^	154	6.6*	5.5–7.4
Sexually transmitted infection	1490	12.4	11.9–13.1
Multiple sexual partners	580	4.8	4.4–5.2
At least one of the above 4 risk factors	4577	38.1	37.3–39.0

While using 12,005 as denominator, the prevalence is 1.3%

^a^Missing 9669 responses hence denominator was 2336*.

### Factors associated with risk factors for HIV

Results of regression analyses are detailed in Table 3. After adjusting for other factors, age, residence, region, sex of household head, working status, marital status and parity remained significantly associated with HIV risk factors.

**Table 3:** Factors associated with risk factors for HIV among women of reproductive age in Sierra Leone as per the 2019 SLDHS

Characteristics	Crude odds ratio COR (95% CI)	P-value	Adjusted odds ratio AOR (95% CI)	P-value
Age				
35 to 49	1		1	
20 to 34	**2.53 (2.32–2.88)**	**< 0.001**	**1.25 (1.05–1.49)**	**0.012**
15 to 19	**13.22 (11.41–15.31)**	**< 0.001**	**1.34 (1.00–1.80)**	**0.052**
Residence				
Rural	1		1	
Urban	**2.34 (2.08–2.62)**	**< 0.001**	**1.49 (1.17–1.89)**	**0.001**
Region				
Western	**1**		1	
Southern	**0.46 (0.39–0.54)**	**< 0.001**	**0.67 (0.50–0.90)**	**0.009**
Northwestern	**0.69 (0.57–0.84)**	**< 0.001**	**1.81 (1.26–2.60)**	**0.001**
Northern	**0.62 (0.53–0.73)**	**< 0.001**	1.25 (0.92–1.68)	0.151
Eastern	**0.51 (0.44–0.59)**	**< 0.001**	0.79 (0.58–1.07)	0.125
Religion				
Islam	1		1	
Christianity	**1.39 (1.25–1.55)**	**< 0.001**	0.98 (0.80–1.20)	0.859
Sex household head				
Male	**1**		**1**	
Female	**2.92 (2.63–3.24)**	**< 0.001**	**1.22 (1.03–1.44)**	**0.024**
Household size				
7 and above	1		1	
Less than 7	**0.69 (0.62–0.76)**	< **0.001**	0.94 (0.81–1.10)	0.435
Working status				
Not working	**1**		**1**	
Working	**0.39 (0.35–0.43)**	**< 0.001**	**1.38 (1.14–1.67)**	**0.001**
Marital status				
Married	**1**		**1**	
Not married	**112.26(91.74–137.37)**	**< 0.001**	**111.17(87.55–141.18)**	**< 0.001**
Education level				
No education	**1**		1	
Primary education	**1.96 (1.68–2.28)**	**< 0.001**	1.09 (0.84–1.41)	0.526
Post-primary education	**4.60 (4.12–5.14)**	**< 0.001**	0.87 (0.73–1.04)	0.123
Wealth Index			–	
Poorest	1			
Poorer	1.12 (0.98–1.29)	0.109		
Middle	**1.40 (1.18–1.65)**	**< 0.001**		
Richer	**2.65 (2.25–3.13)**	**< 0.001**		
Richest	**2.71 (2.31–3.19)**	**< 0.001**		
**Parity**				
5 and above	**1**		**1**	
2–4	**1.70 (1.43–2.02)**	**< 0.001**	**1.26 (1.01–1.57)**	**0.044**
0–1	**9.10 (7.71–10.73)**	**< 0.001**	**1.32 (1.01–1.72)**	**0.043**
Internet access				
No	**1**		1	
Yes	**2.82 (2.50–3.17)**	**< 0.001**	0.94 (0.72–1.23)	0.655
Exposure to radio				
No	**1**		1	
Yes	**1.40 (1.28–1.54)**	**< 0.001**	1.12 (0.95–1.31)	0.171
Exposure to TV				
No	**1**		1	
Yes	**1.89 (1.70–2.09)**	**< 0.001**	1.08 (0.88–1.32)	0.471
Exposure newspapers				
No	**1**		1	
Yes	**2.32 (1.93–2.79)**	**< 0.001**	0.98 (0.67–1.44)	0.918
Visited by fieldworker			–	
No	1			
Yes	0.95 (0.86–1.06)	0.379		
Visited health facility				
No	1		1	
Yes	**0.72 (0.66–0.80)**	**< 0.001**	1.03 (0.89–1.19)	0.710
Permission to access healthcare				
Big problem	1		1	
Not big problem	**1.32 (1.16–1.49)**	**< 0.001**	1.07 (0.87–1.31)	0.534
Distance to health facility				
Big problem	**1**		1	
Not big problem	**1.44 (1.28–1.63)**	**< 0.001**	0.94 (0.76–1.16)	0.555

*AOR* adjusted odds ratio, *COR* crude odds ratio

bold = Significant at p-value < 0.05

Women of 15 to 19 years (AOR = 1.34, 95% CI 1.00–1.80) and those of 20 to 34 years (AOR = 1.25, 95% CI 1.05–1.49) were respectively, 34% and 25% more likely to have HIV risk factors compared to those of 35 to 49 years of age. Urban residents were 49% more likely to encounter HIV risk factors (AOR = 1.49, 95% CI 1.17–1.89). Compared to women in the Western region, those from the South were 33% less likely (AOR = 0.67, 95% CI 0.50–0.90) while those in the Northwest were 81% more likely (AOR = 1.81, 95% CI 1.26–2.60) to have HIV risk factors. Moreover, women from female-headed households (AOR = 1.22, 95% CI 1.03–1.44), and those working (AOR = 1.38, 95% CI 1.14–1.67) were 22% and 38% respectively, more likely to have HIV risk factors, compared to their respective counterparts. Marital status was strongly associated with HIV risk factors; whereby unmarried women were over 111% (AOR = 111.17, 95% CI 87.55–141.18) more likely to have HIV risk factors. In addition, women with parity of 0–1 (AOR = 1.32, 95% CI 1.01–1.72) and 2–4 (AOR = 1.26, 95% CI 1.01–1.57) were 32% and 26% more likely have HIV risk factors, respectively as shown in Table 3.

## Discussion

We conducted a secondary analysis of SLDHS data to estimate the prevalence of HIV risk factors and their socio-demographic associates among women of reproductive age in Sierra Leone. In this study, four factors were considered as potential HIV risk factors, including; non-condom use at last sexual intercourse for unmarried women, engaging in transactional sex, having other sexually transmitted infections (STIs), and having multiple sexual partners. To our knowledge, this is the first study to focus on the burden of HIV risk factors in Sierra Leone.

The study results showed that 38% of the women had encountered at least one of the four risk factors, 27% had non-condom use (the unmarried), 12% had other STIs, while less than 10% had engaged in transactional sex and had multiple sex partners. Although no immediate similar study to compare our results with, the overall prevalence of such risk factors is of significant impact in a low-income country with a high burden of other infectious diseases coupled with a weak health system [3]. Moreover, there is also a lower rate of HIV testing and antiretroviral treatment notably even among pregnant women living with HIV (less than 60%) in Sierra Leon [29]. Although the country currently has a relatively low prevalence of HIV, all the above risk factors collectively pose a threat of increased new infections if not addressed at hand. With yet no effective cure nor vaccine against HIV, preventive measures aimed at reducing new infections mainly through risk-reduction are paramount in the fight against the HIV epidemic. The observed high prevalence of HIV risk factors, therefore, reflects a gap in the HIV/AIDS prevention and control strategies implemented in Sierra Leone, implying a need to strengthen health promotion and sexual behaviour educational programs among reproductive-aged women in the country. However, the reported prevalence, specifically, of non-condom use in this study is less than that reported by the World Health Organisation [30], and that from Tanzania [31], Malawi [32], USA [33] and China [34].

Our study also explored several socio-demographics associated with the burden of HIV risk factors, where age, parity, place of residence, region, sex of household head, marital status, and working status were found to be significant.

Women of younger age were more likely to engage in/encounter HIV risk factors compared to older women in Sierra Leone. This is similar to other studies that have individually reported age to be associated with inconsistent/non-condom use [3, 32], having other STIs [20, 35], transactional sex [20] and multiple sex partners [36]. Younger people are generally curious and common victims of peer influence thus can easily end up engaging in various risk behaviours for not only HIV but also other STIs [20, 35]. Moreover, parity was also significantly associated with HIV risk factors among women, whereby those who had ever produced fewer children were more likely to face HIV risk factors compared to their counterparts of more parity. The possible reason could be that women with no or fewer children are usually younger with less responsibilities and children to look after, so they can easily engage in risky behaviours [20, 36].

The current study results revealed that place of residence is associated with HIV risk factors, where urban women had more chances of encountering HIV risk factors. Due to the appealing and active nature of urban settings, with more entertainment avenues such as bars and clubs, women residing in these places are more exposed to risky behaviours [20]. Moreover, commercialized sexual activities and drug use tend to be more concentrated in urban areas, possibly due to the increasing trends of the urban poor and slum dwellings [20, 37]. Consistently, region was also found to be associated with HIV risk factors, where women in the Northwestern part of the country were more likely to face HIV risk factors. This may be attributed to the differential social-cultural contexts and economic developments in various parts of the country. No wonder, the Northwestern part has a higher concentration of economic development and mining activities, with a generally urban or semi-urban lifestyle [5]. Furthermore, the low employment rate of women in this region lures majority of them to engage in petty trading which exposes them to HIV risk factors during hawking from one community to another [5]. However, our results showed that women in the Southern part were less likely to encounter HIV risk factors yet the region has several fishing communities. The finding deviates from previous studies that have labelled such fishing communities as HIV/AIDS hotspots due to various unique risky behaviours in such communities [3, 38]. Region and place of residence have been reported in other studies to be associated with multiple sex partners [36], inconsistent condom use [39], STIs [39], and transactional sex [37].

Participants from female-headed households were more likely to encounter HIV risk factors compared to those from male-headed households. Consistently, unmarried women also had extremely higher chances of facing HIV risk factors compared to married women. Unmarried women from female-headed households tend to have more freedom from marital obligations and autonomy to do whatever they please, unlike their married counterparts, implying they can easily engage in risky behaviours for survival or economic reasons [36]. Marital status of women has been, disaggregatively, shown in other studies to be associated with transactional sex [37], multiple sex partners [36], and having other STIs [35].

Working status also had an association with HIV risk factors, whereby working women had more chances of encountering HIV risk factors compared to non-working women. Depending on the type and nature of the employment, most informal jobs predispose women to risky behaviours, for instance, working in bars, guesthouses, and sex workers, among others [37, 40]. Furthermore, although regarded as a crucial aspect of women empowerment, employment has been shown to have other negative associates, for example, sexual violence [40]. Working status of women has been reported in previous studies to be associated with having other STIs [35], transactional sex [37], and condom use [41].

## Strengths and limitations

This is the first nationwide analysis that explores the burden of HIV risk factors and the associated socio-demographics, therefore, it can be used as a yardstick and motivation for further studies on the same topic in Sierra Leone and other countries. Secondly, we used the most current nationally representative dataset, thus our findings are generalizable to all women in Sierra Leone. However, our study is not without limitations, for example, the cross-sectional design only enables associations to be established but not causal relationships. There is also a possibility of recall bias due to self-reported answers, and giving false answers due to social desirability. There was also a lack of data on other key HIV risk factors such as alcohol and drug use and the missing data in the transactional sex variable risks underestimating transactional sex prevalence. Moreover, although marital status had extremely large odds ratios, it had a wider confidence interval as well, which could be due to inconsistency in the collected DHS data or other reasons, to which we had no control.

## Conclusions

The study revealed that more than a third of the reproductive aged women in Sierra Leone had encountered at least one HIV risk factor. Age, parity, place of residence, region, sex of household head, marital status, and working status were associated with the prevalence of HIV risk factors in the country. There is thus a need for strengthening HIV/AIDS education and sex behaviour-change communication targeting the young working unmarried women, and those residing in urban areas of the country. In addition, the existing laws and policies in the country should be strengthened to safeguard working women from the associated risks of not only HIV but also other STIs.

## Supplementary Information

**Additional file 1.** Frequency of risk factors for HIV among reproductive aged women in Sierra Leone.

## Data Availability

The data set used is openly available upon permission from MEASURE DHS website (https://www.dhsprogram.com/data/available-datasets.cfm).

## Abbreviations

EA: Enumeration area
AOR: Adjusted odds ratio
CI: Confidence interval
COR: Crude odds ratio
DHS: Demographic Health Survey
SLDHS: Sierra Leone Demographic Health Survey
OR: Odds ratio
SD: Standard deviation
STI: Sexually transmitted infection
AIDS: Acquired immunodeficiency syndrome
HIV: Human Immunodeficiency virus
SPSS: Statistical Package for Social Science

## Availability of data and materials

The data set used in this study is openly available from MEASURE DHS website and the modified data set that was used for the final analysis can be availed upon request from the corresponding author.
