# Supplementary material for: Prevalence of risk factors for human immunodeficiency virus among women of reproductive age in Sierra Leone: a 2019 nationwide survey
Source: BMC Infect Dis. 2022 Jan 17;22:60. doi: 10.1186/s12879-022-07037-7 (PMC8764866; doi:10.1186/s12879-022-07037-7)
Supplement: Supplementary file 1 — Additional file 1. Frequency of risk factors for HIV among reproductive aged women in Sierra Leone. [file 12879_2022_7037_MOESM1_ESM.docx]

**Frequency of risk factors for HIV among reproductive aged women in Sierra Leone**

| **Risky factor** | **Frequency**  **N = 12005** | **Prevalence %** |
| --- | --- | --- |
| None | 7428 | 61.9 |
| One risk factor | 3738 | 31.1 |
| Two risk factors | 751 | 6.3 |
| Three risk factors | 79 | 0.7 |
| Four risk factors | 9 | 0.1 |
